# Differences in international medical graduates’ letters of recommendation by gender in pulmonary and critical care medicine: a cohort analysis

**DOI:** 10.1186/s12909-023-04042-5

**Published:** 2023-01-24

**Authors:** Kaitland M. Byrd, Snigdha Jain, Irada Choudhuri, Başak Çoruh, Jakob I. McSparron, Elizabeth M. Viglianti

**Affiliations:** 1 Department of Internal Medicine Division of Pulmonary and Critical Care, University of Michigan, 2800 Plymouth Road, NCRC building 16, Ann Arbor, MI 48109 USA; 2 Department of Internal Medicine, Section of Pulmonary, Critical Care, and Sleep Medicine, Yale School of Medicine, New Haven, CT USA; 3 Internal Medicine Resident in the Department of Internal Medicine, University of Pittsburgh, Pittsburgh, PA USA; 4 Division of Pulmonary, Critical Care and Sleep Medicine, University of Washington, Seattle, WA USA; 5 Veterans Affairs Center for Clinical Management Research, HSR&D Center for Innovation, Ann Arbor, MI USA; 6 Institute of Health Policy and Innovation, University of Michigan, Ann Arbor, MI USA

**Keywords:** Letters of recommendation, bias, Graduate medical education, Pulmonary and critical care, International medical graduates

## Abstract

**Background:**

International Medical Graduates (IMGs) encounter barriers as they seek to match into fellowship programs in the United States (US). This study’s objective is to determine if there are differences in letters of recommendation written for IMGs compared to U.S. Medical Graduates (USMGs) applying to pulmonary and critical care medicine (PCCM) fellowship programs.

**Methods:**

All applications submitted to a PCCM fellowship program in 2021 were included in this study. The applicant demographics and accomplishments were mined from applications. The gender of letter writers was identified by the author’s pronouns on professional websites. Word count and language differences in the letters were analyzed for each applicant using the Linguistic Inquiry and Word Count (LWIC2015) program. Multivariable linear regressions were performed controlling for applicant characteristics to identify if IMG status was associated with total word count and degree of support, measured by a composite outcome encompassing several categories of adjectives, compared to USMG status.

**Results:**

Of the 573 applications, most of the applicants were USMGs (72%, *N* = 334/573). When adjusting for applicant characteristics, IMG applicants had shorter letters of recommendation (87.81 total words shorter 95% CI: − 118.61, − 57.00, *p*-value < 0.01) and less supportive letters (4.79 composite words shorter 95% CI: − 6.61, − 2.97, *p*-value < 0.01), as compared to USMG applicants. Notably, female IMG applicants had the biggest difference in their word counts compared to USMG applicants when the letter writer was a man.

**Conclusions:**

IMG applicants to a PCCM fellowship received shorter and less supportive letters of recommendation compared to USMG applicants.

**Supplementary Information:**

The online version contains supplementary material available at 10.1186/s12909-023-04042-5.

## Background

Diversity, equity, and inclusion (DEI) efforts in academic medicine have focused on addressing the systemic barriers and challenges encountered by historically underrepresented in medicine (URiM) groups by offering solutions [1]. While attention has predominantly focused on gender and racial minorities, international medical graduates (IMGs) [2, 3], defined as physicians who completed medical school outside of the United States (U.S.) or Canada [4], are often overlooked. Despite encountering numerous barriers and challenges to acquiring a training position in a U.S. program, IMGs make up 25% of the physician workforce and are more likely to care for patients in areas with healthcare shortages and provide care for more vulnerable patient populations [5–7]. These challenges may be implicitly reflected in their letters of recommendation.

Letters of recommendation play a critical role in the application process for trainees and how the letters of IMGs differ from U.S. medical graduates (USMGs) is unknown. Recent work has focused on evaluating letters of recommendation for applicants for systemic gender biases [8–14]. These studies have shown mixed results with women applicants sometimes having longer and more supportive letters as compared to men applicants [15–17]. Additionally, recent studies have suggested that there may be racial disparities in letters of recommendation with URiM applicants having shorter and less supportive letters of recommendation as compared to applicants who identified as white [18–21]. However, these studies did not identify differences for IMGs.

To fill this gap in knowledge, we investigated if letters of recommendation for IMG applicants to a pulmonary and critical care medicine (PCCM) fellowship program differed in length or language as compared to USMGs. Furthermore, we evaluated if the sex differences found in the general applicant population persisted among IMG applicants and if the gender of the letter writer contributed to these differences [21]. We hypothesized that 1) IMG applicants would have shorter letters of recommendation compared to their U.S. counterparts, and 2) female IMG applicant letters would be longer and more supportive as compared to male IMG applicants.

## Method

### Study population

All applications to the University of Michigan’s PCCM fellowship in 2021 were included in the sample.

The applicant’s self-reported race/ethnicity, sex, number of publications, number of presentations, chief medical status (CMR), Alpha Omega Alpha (AOA), and international medical graduate (IMG) status were identified from their applications and added into a de-identified electronic database, REDCap [22]. The race/ethnicity and sex of the applicant were coded to match the groupings used by the ERAS application. We did not adjust for the type of residency training program (e.g., community vs university) as we did not expect the type of program to be associated with gender bias in the letters of recommendation, and letter writers’ hospital affiliations did not always align with those of applicants.

Applicants were identified as an IMG if they completed medical school outside of the U.S. or Canada [4]. Applicants were identified as URiM per the definition used by the Association of American Medical Colleges as “any U.S. citizen or permanent resident who self-identified as one or more of the following race/ethnicity categories (alone or in combination with any other race/ethnicity category): American Indian or Alaska Native; Black or African American; Hispanic, Latino, or of Spanish Origin; or Native Hawaiian or other Pacific Islander.” [23].

The gender of the letter of recommendation writer was identified by how the letter writer was identified on university, hospital, and professional websites (e.g., Doximity and LinkedIn) where the author’s pronouns were available [24]. If no pronouns were able to be identified, the author’s gender was listed as unknown. Since the author’s sex could not be identified, we used pronouns which more likely reflected the author’s gender.

### Data dictionary

In line with previous research, we used the Linguistic Inquiry and Word Count program (LIWC2015; Pennebaker Conglomerates, Inc., Austin, Texas). This program is a word-count based, text analysis program that quantifies language metrics. It has been previously used in multiple studies and fields to study the language used in letters of recommendation [9–12, 20, 25].

The data dictionary we used was based on our prior work, and captures the various adjectives commonly used in letters of recommendation including communal, social-communal, ability, grindstone, positive and negative agentic, research, and standout words [11, 12, 17, 20, 26]. A composite outcome measuring the degree of support was created encompassing grindstone, ability, research, standout, and positive agentic words (Supplemental Table 1).

The letters of recommendation were cleaned and deidentified using Adobe Acrobat Pro DC (Adobe, San Jose, CA). All names, salutations, dates, letterheads, and signatures were removed before processing by LWIC2015.

### Statistical analysis

We used multivariable linear regression to identify if IMG applicants had shorter letters of recommendation as compared to USMG applicants, adjusting for sex, ethnicity, total number of publications, presentations, and CMR status. We did not include AOA status as not all international medical schools have this award.

We also used a multivariable linear regression to identify if IMG applicants had less supportive letters of recommendation, based on the composite outcome as compared to USMG applicants while adjusting for sex, ethnicity, total number of publications, presentations, and CMR status.

We conducted all statistical analysis with Stata software 15.1 (StataCorp).

## Results

Of the 573 applications received in 2021, 72% (*N* = 334/573) of the applicants were USMGs of which the majority were male (64%, *N* = 214/334) and white (57%, *N* = 191/334). Among the IMG applicants, the majority were male (67%, *N* = 161/239) and Asian (46%, *N* = 111/239) (Table 1). There were 2184 letters of recommendation reviewed of which 1280 were for USMGs and 904 were for IMGs. Letter writers were predominantly men (73%, *N* = 1580/2150) for both USMGs and IMGs (Table 1).

**Table 1:** Applicant demographics by IMG status, University of Michigan Pulmonary Critical Care Medicine fellowship cohort, 2021

Variable	IMG Applicant ***N*** = 239	USMG Applicant ***N*** = 334
**Race**		
White: N (%)	51 (21.3)	191 (57.2)
Asian: N (%)	111 (46.4)	92 (27.5)
URiM: N (%)	42 (17.6)	21 (6.3)
Other: N (%)	35 (14.6)	30 (9.0)
**Sex**		
Male: N (%)	161 (67.4)	214 (64.1)
Female: N (%)	78 (32.6)	120 (35.9)
Applicant total publications:^a^ N (IQR)	5 (2,9)	4 (1,6)
Applicant total presentations:^a^ N (IQR)	6 (2,10)	6 (3, 10)
CMR: N (%)	58 (24.3)	97 (29)
Letters of recommendation: median (IQR)	3 (2,4)	3 (2,4)
**Word count per letter of recommendation: mean (SD)**	463.1 (277.5)	565.3 (360)
**Letter writers**	*N* = 904	*N* = 1280
Woman: N (%)	209 (23.1)	361 (28.2)
Man: N (%)	682 (75.4)	898 (70.2)
Unknown: N (%)	13 (1.4)	21 (1.6)

*IMG* International medical graduate, *USMG* United States medical graduate, *URiM* Underrepresented in medicine defined as per AAMC, *CMR* Chief medical resident, *SD* Standard deviation, *IQR* Interquartile range

^a^Applicant research activities included peer viewed abstracts, posters, oral presentations; book chapters and online publications

USMG applicants received longer letters of recommendation (total word count: 565, standard deviation [SD]: 360) as compared to IMG applicants (total word count: 463, SD: 277). IMG applicants received shorter letters of recommendation from both men and women letter writers as compared to USMG applicants and their letters included fewer adjectives in all categories used in the LWIC dictionary (Table 2).

**Table 2:** Average word counts by the sex of the applicant and the gender of the letter writer, by IMG status

Variables	Gender of Letter Writer		Gender of Letter Writer	
	IMG Applicant		USMG Applicant	
Composite word count: Mean (SD)	Man	Woman	Man	Woman
Sex of Applicant: Male	20.75 (13.50)	28.29 (16.10)	26.03 (20.28)	29.92 (22.64)
Female	22.47 (15.54)	29.16 (20.24)	29.27 (22.01)	33.13 (26.07)
Communal Words (word counts: mean, SD)				
Sex of Applicant: Male	3.26 (2.81)	4.48 (3.80)	3.85 (3.73)	4.56 (4.10)
Female	3.51 (2.87)	4.43 (4.35)	4.05 (3.95)	4.60 (5.11)
Grindstone Words (word counts)				
Sex of Applicant: Male	3.84 (3.17)	5.37 (4.44)	4.47 (3.88)	5.59 (5.27)
Female	3.87 (3.14)	5.57 (4.42)	4.96 (4.30)	5.83 (5.84)
Social Communal (word counts)				
Sex of Applicant: Male	0.65 (0.99)	0.77 (0.99)	0.68 (1.00)	0.96 (1.29)
Female	0.67 (0.91)	0.97 (1.41)	0.87 (1.13)	0.94 (1.12)
Positive Agentic Words (word counts)				
Sex of Applicant: Male	5.44 (3.82)	7.15 (4.74)	6.75 (6.01)	7.55 (5.87)
Female	5.81 (4.72)	7.29 (5.14)	7.49 (6.23)	8.12 (6.56)
Negative Agentic Words (word counts)				
Sex of Applicant: Male	0.01 (0.13)	0.01 (0.09)	0.01 (0.10)	0.02 (0.16)
Female	0.02 (0.17)	0.00 (0.00)	0.00 (0.05)	0.02 (0.12)
Ability words (word counts)				
Sex of Applicant: Male	4.31 (3.48)	6.05 (4.16)	5.07 (4.59)	5.41 (4.56)
Female	4.47 (3.82)	6.05 (5.48)	5.71 (4.36)	6.10 (5.38)
Standout words (word counts)				
Sex of Applicant: Male	3.81 (3.32)	4.38 (3.04)	4.84 (4.57)	4.97 (4.86)
Female	4.07 (3.28)	4.99 (4.51)	5.62 (5.90)	5.44 (5.20)
Research (word counts)				
Sex of Applicant: Male	3.56 (4.60)	5.35 (5.48)	4.90 (5.85)	6.41 (6.90)
Female	4.24 (4.81)	5.26 (4.94)	5.49 (6.27)	7.64 (8.88)

*SD* Standard deviation

When adjusting for applicant demographics (e.g., sex, race/ethnicity) and accomplishments (e.g., CMR status, number of publications and presentations), IMG applicants had shorter letters of recommendation (87.81 total words shorter 95% CI: − 118.61, − 57.00, *p*-value < 0.01) and less supportive letters (4.79 composite words shorter 95% CI: − 6.61, − 2.97, *p*-value < 0.01) as compared to USMG applicants (Table 3).

**Table 3:** Fellowship applicant demographics and accomplishments associated with letter of recommendation length and degree of support

Variables	Total words		Composite Words	
	Coefficient (95%CI)	*p*-value	Coefficient (95%CI)	*p*-value
Male applicant (compared to female)	−32.45 (−60.77, −3.13)	0.03	−2.21 (−3.94, −0.48)	0.01
IMG (compared to USMG)	−87.81 (−118.62- -57.00)	< 0.01	−4.79 (− 6.61- -2.97)	< 0.01
Total number of publications	0.00 (−2.58–2.58)	0.99	0.13 (−0.03–0.28)	0.11
Total number of presentations	4.80 (2.17–7.42)	< 0.01	0.24 (0.09–0.40)	< 0.01
Race (as compared to White)				
Asian	−30.09 (−63.39–3.22)	0.08	−1.41 (− 3.37–0.56)	0.16
URiM	− 34.03 (−83.14–15.08)	0.17	− 1.15 (− 4.05–1.75)	0.44
Other	8.47 (− 39.10–56.05)	0.73	−0.51 (− 3.31–2.30)	0.72
CMR: Yes (compared to No)	−10.61 (−42.27–21.04)	0.51	− 0.94 (− 2.81–0.93)	0.32
Man letter writer (compared to woman)	−85.42 (− 116.82- -54.02)	< 0.01	−5.14 (− 6.99- -3.29)	< 0.01

*CI* confidence interval, *URiM* underrepresented in medicine, *CMR* chief medical resident

## Discussions

### Key findings

To our knowledge, no prior studies have evaluated the differences in letters of recommendation for IMGs as compared to USMGs applying to PCCM fellowship programs. We found that IMG applicants had shorter and less supportive letters of recommendation as compared to USMGs. Notably, female IMG applicants had the biggest difference in the length of their letters of recommendation as compared to USMG applicants when the letter writer was a man. Lastly, women letter writers wrote longer and more supportive letters as compared to men.

### Relationship to previous studies

Our work begins to shed light on the differences that exist in the letters of recommendation for IMGs as compared to USMGs. Our findings demonstrate that IMGs have less supportive and shorter letters as demonstrated by the less frequent use of every adjective in our LWIC dictionary. While attention has focused on reducing biases in female applicant letters of recommendation, less attention has been given to differences in letters of recommendation for IMGs. These differences could suggest structural bias against IMGS, resulting in career development delays with downstream implications on the physician workforce [27–29].

The growing attention to address systematic barriers that impact DEI efforts in medicine has resulted in several studies evaluating bias in letters of recommendation. Historically this work was focused on the gender and URiM status of applicants, examining length of letters, discussions of research, and types of adjectives used by letter writers [20, 30]. While more recent work suggests that the trend may be changing for women applicants to PCCM, radiation oncology, and surgery, no work has evaluated the role of letters of recommendation in exacerbating the challenges faced by IMGs as they pursue specialty training and how the language in their letters may hinder them in their pursuits [15, 20, 21].

### Study implications

Research aimed at meeting academic medicine’s stated goal of improving DEI also needs to focus on addressing the existing biases within letters of recommendation for IMGs. IMGs make up approximately 25% of the physician workforce in the U.S. and are more likely to care for patients in underserved areas [31]. IMGs are also less likely to match into residency programs [32]. One step toward improving the representation of women, IMGs, and URiM fellows is addressing the disparities in language within letters of recommendation and educating letter writers of these biases.

Historically disadvantaged groups have had to work “twice as hard and twice as long” to even be considered acceptable for a position [13, 33]. This phenomenon has been described in the challenges women and other URiM physicians have encountered in medicine throughout their academic career and has been a focus of why the content of letters of recommendation matters so much more [34]. Our study sheds light on the unfortunate reality of how disparate the letters of recommendation are for IMGs. Based on the implicit biases held against disadvantaged groups, IMGs would not only need similar length and supportive letters of recommendation, but their letters of recommendation would need to be longer and more supportive for them to be considered “acceptable” [27–29, 35, 36]. Until we establish clear, comprehensive evaluations of candidates that are not heavily dependent on letters of recommendation, personal and systematic implicit biases will continue to impact residents’ ability to match into the specialty of their choice and hinder efforts to improve DEI within academic medicine.

One step toward reducing the impact of systematic biases in letters of recommendation for fellowship applicants is for programs to move away from using letters of recommendation to evaluate applications.

### Strength and limitations

There are several limitations to our study. First, the LWIC2015 program relies on word counts and does not offer insight into the context in which the words are being used. Second, this is a single site study and only includes applicants to an academic PCCM program, making it difficult to generalize beyond the subfield. Third, we were unable to distinguish between U.S. citizens who attended medical school abroad and IMGs who do not have U.S. citizenship. Fourth, it remains unknown if the length of the letters of recommendation truly matters in the selection process of applicants. However, having letters which encompass more supportive adjectives likely reads more favorably to selection committees. Finally, we did not adjust for university versus community-affiliated status of the residency programs – since IMGs are more likely to train at community-affiliated programs [2], it is possible that the training and experience of faculty writing the letters of recommendation might influence the content and length of the letters.

## Conclusion

IMGs applicants as compared to USMG applicants to an academic PCCM program received shorter and less supportive letters of recommendation. Further work is needed to determine if these findings vary based on the type of residency program or the applicant’s immigration status.

## Supplementary Information

**Additional file 1: Table 1.** Data Dictionary for LWIC2015

## Data Availability

The datasets used and analyzed during the current study are available from the corresponding author on reasonable requests.

## Abbreviations

IMG: International medical graduate
USMG: United States medical graduate
PCCM: Pulmonary and critical care medicine
DEI: Diversity, equity, and inclusion
URiM: Underrepresented in medicine
CMR: Chief medical resident
AOA: Alpha Omega Alpha
SD: Standard deviation
