# Supplementary material for: Differences in international medical graduates’ letters of recommendation by gender in pulmonary and critical care medicine: a cohort analysis
Source: BMC Med Educ. 2023 Jan 24;23:58. doi: 10.1186/s12909-023-04042-5 (PMC9875522; doi:10.1186/s12909-023-04042-5)
Supplement: Supplementary file 1 — Additional file 1: Table 1. Data Dictionary for LWIC2015 [file 12909_2023_4042_MOESM1_ESM.docx]

**Supplemental Appendix**

**Table 1**: Data Dictionary for LWIC2015

| **Communal** | **Grindstone** | **Social Communal** | **Positive Agentic** | **Negative Agentic** | **Ability** | **Standout** | **Research** |
| --- | --- | --- | --- | --- | --- | --- | --- |
| affectionate | assiduous | babies | able | daring | ability | amazing | abstract* |
| agreeable | busy | brothers | ambitious | forceful | accomplished | best | author* |
| approachable | careful | children | assertive | outspoken | adept | excellent | conference* |
| calm | conscientious | colleagues | bright | provocative | adroit | exceptional | contribution |
| caring | dedicated | dad | bright future | reserved | analytical | extraordinary | data |
| cheerful | dedication | family | capable |  | aptitude | extremely | discover |
| compassion* | dependable | gentleman | clever |  | beyond expected level | fabulous | experiment |
| congenial | determination | husband | competent |  | brain | impeccable | finding* |
| conscientious | diligent | mother | confidence |  | bright | impressive | fund |
| delightful | discipline | sister | confident |  | brilliant | magnificent | grant |
| devoted | effort | wife | determined |  | capacity | most | journal* |
| eager* | focused |  | dominant |  | clever | outstanding | manuscript* |
| easy to work | hard-working |  | efficient |  | creative | remarkable | method |
| empathy | hardworking |  | excel |  | expert | superb | poster* |
| empathetic | hard working |  | exceptional |  | flair | supreme | presentation* |
| enthusiasm | industrious |  | exemplary |  | genius | star | project* |
| enthusiastic | methodical |  | independent |  | gift | terrific | publication* |
| friendly | meticulous |  | industrious |  | inherent | unique | publish* |
| generous | motivated |  | intellectual |  | innate | unmatched | research |
| happy | motivation |  | intelligent |  | innovative | unparalled | result |
| helpful | organized |  | leader |  | inquisit* | wonderful | scholarship |
| honest | persist |  | leadership |  | insightful |  | science |
| humble | professional |  | passion* |  | instinct | best | studies |
| humility | reliable |  | persistent |  | intelligent | excellent | study |
| interpersonal | responsib* |  | powerful |  | knack | exceptional | test |
| kind | tenacious |  | problem solver |  | knowledge* | extraordinary | theory |
| kind* | thorough |  | rising star |  | natural |  |  |
| mature | trust |  | solid |  | proactive |  |  |
| likeable | work |  | strong |  | proficient |  |  |
| nurturing |  |  | superior |  | propensity |  |  |
| outgoing |  |  | trustworthy |  | skill* |  |  |
| personable |  |  | well-rounded |  | smart |  |  |
| pleasant |  |  |  |  | talent* |  |  |
| polite |  |  |  |  |  |  |  |
| positive |  |  |  |  |  |  |  |
| rapport |  |  |  |  |  |  |  |
| respectful |  |  |  |  |  |  |  |
| sensitive |  |  |  |  |  |  |  |
| soft spoken |  |  |  |  |  |  |  |
| sympathetic |  |  |  |  |  |  |  |
| tactful |  |  |  |  |  |  |  |
| team player |  |  |  |  |  |  |  |
| thoughtful |  |  |  |  |  |  |  |
| warm |  |  |  |  |  |  |  |
| well-liked |  |  |  |  |  |  |  |

*words that can have multiple ending (i.e. compassion, compassionate, compassionately)
